# Skeletal involvement of hairy cell leukemia

**DOI:** 10.1002/ccr3.1635

**Published:** 2018-07-02

**Authors:** Eric Durot, Anne Quinquenel, Jean‐Charles Kleiber, Martine Patey, Arnaud Bazin, Alain Delmer

**Affiliations:** ^1^ Service d’Hématologie Clinique Centre Hospitalier Universitaire Hôpital Robert Debré Reims France; ^2^ UFR Médecine Université Reims Champagne‐Ardenne Reims France; ^3^ Service de Neurochirurgie Centre Hospitalier Universitaire Hôpital Robert Debré Reims France; ^4^ Laboratoire de Biopathologie Centre Hospitalier Universitaire Hôpital Robert Debré Reims France

**Keywords:** hairy cell leukemia, hematology, skeletal involvement, skull

## Abstract

Hairy cell leukemia (HCL) is a rare B‐cell lymphoproliferative disorder. Skeletal involvement is an unusual manifestation of HCL, complicating the course of the disease in approximately 3% of patients. We describe a case of skull involvement by HCL, a localization rarely reported in the literature.

A 45‐year‐old woman presented with a 2‐month history of painless skull tumefaction. She was diagnosed with hairy cell leukemia (HCL) 18 months earlier following incidental finding of mild cytopenias and abnormal circulating lymphoïd cells with cytoplasmic projections. Bone marrow aspirate was hypocellular with 10% of monotypic lymphoïd B cells expressing CD20, CD11c, CD25, and CD103. The search for BRAF V600E mutation was positive. At that time, no specific treatment was given since cytopenias were moderate. Physical examination revealed median parieto‐occipital tumefaction. There was no splenomegaly or peripheral lymphadenopathy. Cranial CT scan and MRI showed a lytic parieto‐occipital lesion with intracranial and subcutaneous extension (Figure 1A,B). The patient underwent surgical subtotal resection of the mass. Histological examination disclosed a massive infiltration of cranial vault with soft tissue extension by CD20(+) CD43(−) DBA44(+) and BRAF(+) lymphoïd cells (Figure 1C,D). The diagnosis of skull involvement by the known HCL was thus retained. The leukocyte count was 2.9 × 10^9^/L with marked neutropenia (0.7 × 10^9^/L), hemoglobin level was 9.6 g/dL, and platelets were 93 × 10^9^/L. Treatment by cladribine (2‐CdA) was therefore initiated and led to ongoing complete hematological response after 2‐year follow‐up.

**Figure 1:**
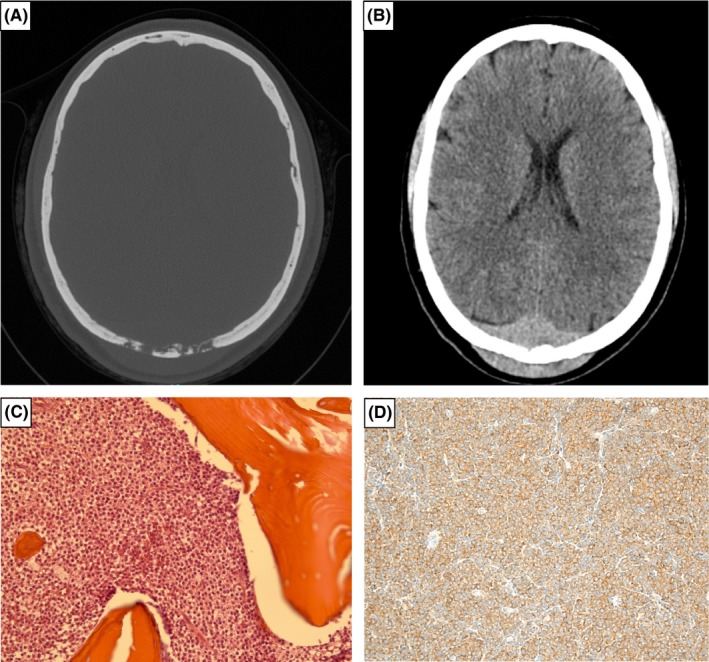
Cranial CT scan showing a lytic lesion (A) with intracranial and subcutaneous infiltration (B). Biopsy section of the mass revealed diffuse infiltration by lymphoïd cells (C, hematoxylin and eosin stain; original magnification 20×) with immunohistochemistry staining for BRAF (D, original magnification 20×)

Hairy cell leukemia is a rare B‐cell lymphoproliferative disorder characterized by the presence of lymphoïd cells with typical morphological and phenotypic features accumulating in the bone marrow and the spleen. Patients usually present with cytopenias and splenomegaly. Treatment relies on purine analogs with remarkable response rates. Skeletal involvement is an unusual manifestation of HCL, complicating the course of the disease in approximately 3% of patients and rarely observed at presentation.^1^, ^2^ The lesions, mainly osteolytic and painful, are preferentially distributed in the axial skeleton and proximal long bones, but very few cases of skull involvement have been reported. Most patients received local radiation therapy (RT) but whether or not RT is useful in addition to purine analogs remains uncertain.

## CONFLICT OF INTEREST

None declared.

## AUTHORSHIP

ED and AD: participated in care of the patient and wrote the manuscript; AQ, JCK, and AB: participated in care of the patient and revised the manuscript content; MP: performed histological analysis and revised the manuscript content. All the authors approved the final version of the manuscript.
